# Casting Welding from Magnesium Alloy Using Filler Materials That Contain Scandium

**DOI:** 10.3390/ma15124213

**Published:** 2022-06-14

**Authors:** Vadym Shalomeev, Galyna Tabunshchyk, Viktor Greshta, Kinga Korniejenko, Martin Duarte Guigou, Sławomir Parzych

**Affiliations:** 1 National University Zaporizhzhya Polytechnik, 64 Zhukovs’ Kogo Street, 69063 Zaporizhzhya, Ukraine; greshtaviktor@gmail.com; 2 Faculty of Material Engineering and Physics, Cracow University of Technology, Jana Pawła II 37, 31-864 Cracow, Poland; kinga.korniejenko@pk.edu.pl (K.K.); slawomir.parzych@pk.edu.pl (S.P.); 3 Department of Engineering and Technology, Universidad Católica del Uruguay, B de Octubre 2738, Montevideo 11600, Uruguay; martin.duarte@ucu.edu.uy

**Keywords:** magnesium alloy, filler material, welding, scandium, microstructure modification, mechanical properties

## Abstract

Based on the results achieved in systematic studies of structure formation and the formation of multicomponent phases, a scandium-containing filler metal from system alloy Mg-Zr-Nd for welding of aircraft casting was developed. The influence of scandium in magnesium filler alloy on its mechanical and special properties, such as long-term strength at elevated temperatures, was studied by the authors. It is established that modification of the magnesium alloy with scandium in an amount between 0.05 and 0.07% allows a fine-grained structure to be obtained, which increases its plasticity up to 70% and heat resistance up to 1.8 times due to the formation of complex intermetallic phases and the microalloying of the solid solution. Welding of the aircraft castings made of magnesium alloy with scandium-containing filler material allows obtaining a weld with a dense homogeneous fusion zone and the surrounding area without any defects. The developed filler material for welding surface defects (cracks, chips, etc.) formed during operation on aircraft engine bodies makes it possible to restore cast body parts and reuse them. The proposed filler material composition with an improved set of properties for the welding of body castings from Mg-Zr-Nd system alloy for aircraft engines makes it possible to increase their reliability and durability in general, extend the service life of aircraft engines, and obtain a significant economic effect.

## 1. Introduction

The development of the aerospace industry results in a constant increase in the complexity of various mechanisms and assemblies, which leads to an increase in their metal intensity [1,2]. One of the solutions to this problem is the use of lightweight materials with a high complex of special mechanical properties [3,4]. Cast magnesium alloys are one of the lightest structural materials, with a density of approximately 1.73 g/cm^3^; their low density is approximately 25% that of steel and 60% that of aluminum [4,5]. Furthermore, they have a high specific strength [6]. This makes them a perfect material for the manufacturing of aerospace engineering and the aircraft industry [6,7]. Magnesium alloys must have good casting properties, a high complex of mechanical characteristics, heat resistance, and good weldability [7,8]. Another advantage of magnesium is its recyclability [8,9,10].

The complexity of aerospace structures and imperfect casting technology require the usage of additive welding technologies [11,12]. Therefore, there is evident progress in the technological trends that combine casting and welding technologies [13,14]. At the same time, when using magnesium alloys, it is necessary to take into account their following characteristics: rather low thermal conductivity, high ignition temperature, increased deformation and stresses during welding due to the high linear expansion coefficient, high gas absorption, the possibility of crystallization crack formation due to fusible eutectics, and large crystallization temperature interval [15,16].

However, magnesium alloys have a lot of advantages, and their applications are also connected to some challenges, including susceptibility to corrosion, low absolute strength, difficulty with deformation, weak chemical stability, flammability, and high price [9,17,18]. Therefore, for engineering and structural applications, magnesium alloys are modified to strengthen their weaker properties without sacrificing their key features [9,19]. The possible methods of modification are alloying, heat treatment, a new preparation method, manufacturing processes, and the application of some additives to magnesium alloys [18,19]. The main objective of these improvements is usually to improve the chemical stability and mechanical properties of magnesium alloys [18,20,21]. The most popular reinforcement that has been studied in recent years is nano-sized particles [9,19] and rare elements [18,20,22]. The additives of rare elements improve the strength and corrosion resistance of magnesium alloys, despite changing the structure of the metal [5,18,20]. However, some investigations have been provided in this area, although this topic requires further research [7,18]. One of the promising additives in this group is scandium [20,23,24].

Alloys of the Mg-Zr-Nd system are promising materials for the production of various molded parts in aerospace engineering [25,26]. They have a fine-grained structure, high physical and mechanical properties, and heat resistance, but have a tendency to crystallize cracks [26,27]. Usually, products made of such magnesium alloys by TIG welding are welded by a non-consumable electrode in an inert gas medium with the use of filler material. The welding filler material is made from the same alloy. This welding technique does not produce a high-quality weld. Defects such as micro-cracks and micro-pores can appear in the weld and seam area [25,28].

The most effective approach in solving the task of increasing the technological strength of welded joints of cast parts made of magnesium alloys is the development of new additive scandium-containing materials with an increased level of mechanical properties and heat resistance, which provide a decrease in the level of residual stresses and the prevention of cracking [29]. The positive effect of scandium is provided on the one hand by the formation of complexly alloyed intermetallic phases, which are additional crystallization centers and refine the grain. On the other hand, the alloy hardening occurs due to the scandium microalloying of the solid solution. Based on global experience, scandium alloys have a high level of mechanical properties and heat resistance; in addition, the welded joints of these alloys are characterized by improved corrosion resistance [23,30]. The usage of scandium alloys is currently constrained by their relatively high cost. However, the requirement to reduce the weight and metal consumption of structures allows the prediction of their widespread use in the near future and ensures the creation of a unified welding technology of complex alloyed magnesium alloys with an improved set of properties [31,32,33]. The solution to this urgent problem is the central task of research.

The purpose of the research is to develop a filler material for welding surface defects (cracks, chips, etc.) formed during operation on aircraft engine cases. This enables the restoration of cast body parts and their reuse to ensure reliable and durable operation.

## 2. Materials and Methods

### 2.1. Materials

The magnesium alloy, with a nominal composition given in Table 1, was smelted in an IPM-500 induction crucible using serial technology.

The refining of the alloy was performed with flux VI-2 in a distribution furnace with batch selection of the melt, which introduced increasing additives of scandium (from 0.05 to 1.0% wt.) Magnesium–scandium ligature (10% Sc, 90% Mg) and standard samples for mechanical tests Ø 12 mm in sand–clay form. The samples were heat treated in Bellevue and PAP-4M furnaces according to the mode: hardening from 415 ± 5 °C, holding time 15 h, cooling in air and aging at 200 ± 5 °C, holding time 8 h, cooling in air.

### 2.2. Research Methods

Temporary tensile strength and elongation of the samples were determined on a P5 rupture machine P5 at room temperature. The long-term strength at different temperatures was determined on an AIMA 5-2 bursting machine(Russia) on samples Ø 5 mm.

To study the weldability of metal heat-treated plates measuring 200 × 100 × 10 mm of the experimental magnesium alloy, we welded with filler material in the form of cast electrodes Ø 8 × 200 mm from the same alloy containing scandium (between 0.06 and 0.07% wt.). Welding was performed with a non-expendable tungsten electrode in argon medium using a welding transformer TD-500 (Kharkiv, Ukraine), oscillator OSPP-3 (Kiev, Ukraine), ballast rheostat RB-35 (Ukraine), and an argon flow rate between 14 and 18 l/min. For mechanical tests, cylindrical samples Ø 5 mm were made so that the boundary of the transition zone from the weld to the base metal was in the middle of the samples. This limit was detected by etching with a reagent consisting of 1% nitric acid, 20% acetic acid, 19% distilled water, and 60% ethylene glycol.

The quality of the weld was monitored by X-ray. Micro-X-ray spectral analysis of the phases was performed on an electron microscope JSM-6360LA (Tokyo, Japan). The microstructure of the metal was studied under a microscope “Neophot 32”. The microhardness of the structural components of the alloy was determined on a microhardness tester company “Buehler” at a load of 0.1 N.

## 3. Results

### 3.1. Microstructure Analysis

**Figure 1:**
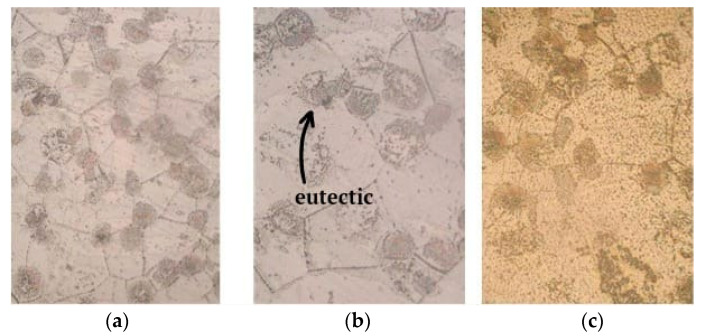
Microstructure of the heat-treated alloy ML10, magnification 500×: (**a**) without Sc; (**b**) 0.05% Sc; (**c**) 1.0% Sc.

The microstructure of the initial heat-treated alloy was a δ-solid solution with the presence of a eutectic (δ + γ (MgZr12Nd)) spherical shape (Figure 1a). With increasing scandium content in the alloy, the size of the eutectic increased (Figure 1b,c).

**Figure 2:**
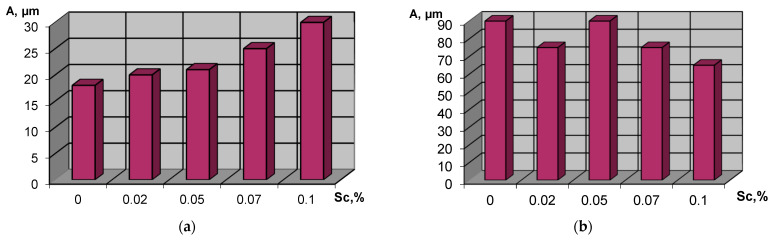
The average size of the structural components: (**a**) of the magnesium alloy with different scandium content: a-(δ + γ)–phase (a eutectic), (**b**) -(δ)–phase (solid solution).

Thus, when more than 0.07% Sc was introduced into the melt, the size of the eutectic regions increased approximately three times compared to the initial alloy, while the grain size of the matrix (δ) was practically at the same level (Figure 2). Such an increase in the size of the eutectic phase is due to the presence of an additional amount of fine intermetallic compounds containing scandium.

### 3.2. Microhardness

The microhardness of the structural components of the experimental alloy increased with increasing scandium content, both before and after heat treatment. Moreover, after heat treatment, there was an increase in the microhardness of the matrix and a decrease in the values of the hardness of the eutectic (Table 2), which is associated with the redistribution of scandium between structural components.

### 3.3. Micro-X-ray Spectral Analysis

Micro-X-ray spectral analysis of the eutectic showed that it has an elemental composition, as given in Table 3.

The scandium content in the eutectic was between 1.5 and 2.0 times higher than in the δ-solid solution. Thus, doping of the magnesium alloy with scandium leads, first of all, to the saturation of the eutectic phase. This is confirmed by the increase in the microhardness of the eutectic with the increase in scandium additives in the alloy.

When the scandium content in the alloy is not more than 0.05%, there was an increase in both mechanical and heat-resistant properties due to the microalloying of structural components. A further increase in the concentration of scandium in the metal led to an increase in the intermetallic phase, redistribution of excess intermetallics at the grain boundary, and reduced the physical and mechanical characteristics of the material (Table 4).

### 3.4. The Study the Weldability

The positive effect of scandium on the long-term strength of the alloy at elevated temperatures was noted. The increase in the content of scandium in the alloy, leading to microalloying of its structural components, helped to increase its heat resistance. Increasing the temperature during the long-term strength test reduced the time to failure. Fine intermetallic particles were released unevenly, forming areas of a striped structure with high microhardness, leading to the destruction of the metal.

In the structure of samples containing more than 0.07% scandium, coarse boundary inclusions were observed, leading to the rapid destruction of samples during testing. Thus, the content between 0.05 and 0.06% of the scandium mass in the experimental magnesium alloy is optimal for the manufacture of filler material with improved properties.

Qualitative indicators of Mg-Zr-Nd alloy castings welded with filler material with scandium content between 0.05 and 0.06%wt., including the weld and close-seam region, were studied. The structure of the seam area is characteristic of the heat-treated alloy. In the structure of the weld, δ-solid solution and γ-phase located along the grain boundaries were observed in the form of light gray emissions.

The dimensions of the structural components of the weld in comparison with the base metal were significantly smaller (Table 5), and the microhardness of the weld was slightly higher.

Mechanical tests of samples with a weld showed that the destruction of the metal took place in the near-seam zone because the weld metal had a fine-grained structure due to accelerated crystallization, which positively contributed to the improvement of the quality of the test metal.

An industrial test of the additive alloy with scandium at welding was carried out during the welding of magnesium alloy body casting elements. There was conducting welding of elements of fuel pump housings, low-pressure compressor housings, and gearbox housings, which are integral parts of aircraft engines. After, these areas were cleaned until the complete removal of defects and thoroughly degreased. The prepared products were placed in a thermal oven, heated to 200 °C, kept at this temperature for 6 h, removed from the oven, and welded. Welding was performed by an argon-arc method using a non-consumable tungsten electrode and a welding transformer TD-500, oscillator OSPP-3, and ballast rheostat RB-35. Rods Ø 8 × 200 mm made of the studied magnesium alloy with scandium were used as the filler material. The welded parts were again loaded into a thermal oven and cooled together with the oven to a temperature not exceeding 120 °C. The welding areas were monitored and cleaned to obtain the required geometric dimensions.

**Figure 3:**
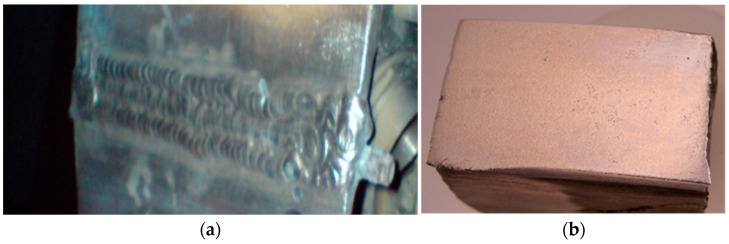
Magnesium alloy body casting element after welding: (**a**) magnification 0.1×; (**b**) macrograph.

Welded body casting elements were cut at the welding sites to determine the quality of the metal. Metallographic and X-ray inspection showed that the weld and the base metal (Figure 3) had a dense homogeneous structure without defects. The weld microstructure consisted of a δ-solid solution and intermetallic phase containing Sc, Zr, and Nd.

**Figure 4:**
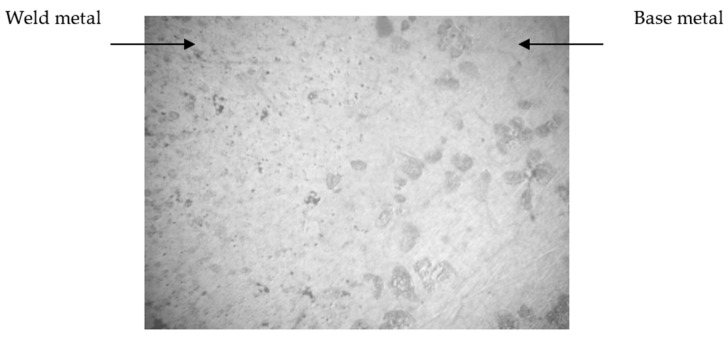
Microstructure of magnesium alloy near the weld zone, magnification 200×.

In this case, the size of the structural components in the weld was much smaller than in the main metal (Figure 4). The weld metal did not contain any defects (micropores, microcracks, etc.) and corresponded to the base metal in terms of mechanical properties.

The developed technology of welding castings from a magnesium alloy enables the restoration of difficult case details of aircraft engines in which qualitative indicators satisfy the requirements of regulatory and technical documentation.

## 4. Discussion

This investigation showed the possibility of using magnesium alloys to produce elements for the aerospace industry. The additive of scandium optimized the microstructure of the alloy and increased its properties. Other studies in the literature confirm the presented results, especially the strong influence of scandium on the microstructure of magnesium alloys [23]. Li et al. [23] showed that a scandium additive of up to 1% wt. refined the grain sizes of the material. They defined the main mechanism of this phenomenon as the heterogeneous nucleation mechanism [23].

Additionally, the mechanical properties of the magnesium alloy with scandium were changed, including the improvement in yield [23,34]. The investigation provided by Li et al. [34] confirmed the improvement in the Vickers hardness magnesium alloys with the addition of scandium compared to pure magnesium alloys. One of the implications of this research is the conclusion that the addition of scandium could enhance the wear resistance of magnesium alloys [34].

Other research shows that scandium can improve magnesium alloy’s properties, including corrosion resistance [35,36]. This fact can support the application of this material not only in the aerospace industry but also in other areas, such as implant applications [37,38].

It is worth noting that a similar mechanism was also observed in aluminum–magnesium alloys as well as other alloys that contain magnesium [39,40].

Another challenge is to study scandium as an additive in the context of material weldability, which is not a trivial issue [8,41]. In the literature, there are only a few studies in this area. Most of them are related to the development of additive manufacturing technologies [5,42,43]. The obtained results show that the addition of scandium could have a positive influence on the development of welding technology as well as additive manufacturing for magnesium alloys and help overcome challenges connected with processing magnesium alloys [5,44].

Magnesium-based alloys that contain zirconium and neodymium, which form heat-resistant intermetallic phases and provide the required service characteristics of the alloy at elevated temperatures, are widely used for the production of heat-resistant magnesium castings in aircraft engines. The range of cast parts made from this alloy is quite diverse and involves a combination of different technologies for their manufacturing (casting, welding, heat treatment, etc.). Welding of the products made of the Mg-Zr-Nd alloy system is carried out with the use of a filler material and an alloy base. In this case, parts of the complex configuration are welded poorly; often, microcracks are formed in the welding spots, which require re-welding. After several unsuccessful welding operations, this product is rejected and sent for remelting. One way to solve this problem is to develop a new composition of a filler material with higher mechanical and special properties compared to the base metal. Therefore, it is important to study the effect of scandium on the structure and properties of the alloy of the Mg-Zr-Nd system, which already has heat-resistant phases (MgZr) 12Nd in its composition, which will improve the physical and mechanical characteristics of the metal in the place of welding.

The research was carried out in two stages:

1. The development of a magnesium-based additive alloy with optimal scandium content to ensure improved mechanical properties and heat resistance.
2. The investigation of the structure and properties of the base metal and the weld on samples welded with the developed scandium-containing filler material.

The structure and properties of a magnesium alloy containing neodymium and zirconium and additionally modified with scandium in an amount of up to 1.0% were studied. Metallographic analysis of the investigated metal showed that additives in the alloy contributed to an increase in the size of the spherical areas of the precipitation of eutectoid. Thus, when more than 0.07% Sc was introduced into the melt, the size of the eutectoid areas increased approximately four times compared to the standard alloy, while the size of the δ-phase was approximately at the same level.

Thermal treatment contributes to the homogeneity of the alloy due to the redistribution of elements between the dendrite axes and interaxial spaces, as well as additional alloying of the matrix due to the diffusion of elements from the boundary precipitations of the (MgZr)12Nd phase.

Micro X-ray diffraction analysis showed that the spherical regions were enriched, mainly with zirconium, neodymium, and scandium. In modified alloys, the scandium content in the spherical regions of δ + (MgZr)12Nd eutectoid excretions was ~1.5–2.0 times higher than in the δ-solid solution.

When the content of scandium in the alloy was lower than 0.3%, grain refinement was observed. A further increase in the filler modifier (up to 1.0% Sc) led to an increase in the size of the micrograins up to 160 µm (in the range of 0.02–0.3% Sc, the size of the micrograins is ~75 µm).

Studies on the heat resistance showed that the samples heated to temperatures of 150–250 °C showed the decomposition of eutectoid. Analysis of the microstructures showed that in the process of exposure to temperature and prolonged exposure along with the decomposition of the eutectoid, its dissolution in the matrix with the subsequent release of intermetallic phases of the (MgZr)12Nd type was the form of finely dispersed particles. At the same time, the fine intermetallic particles were isolated irregularly, forming areas of a banded structure characterized by an increase in microhardness values.

It was established that a more complete disintegration of the eutectoid phase was promoted by the holding time at a given temperature, as well as by the stress value. At temperature 270 °C, coarsening of the structure was observed due to intensive allocation of intermetallides, in particular, on borders of grains, which explains the sharp decrease in the heat resistance of the material. The coarsest boundary separation was detected in the structure of samples containing more than 0.07% Sc, leading to the rapid destruction of samples during the long-term strength test.

The δ-solid solution microhardness of the standard alloy (before heat treatment) was more than three times lower than the microhardness of the isolations in the spherical eutectoidal regions. After heat treatment, an increase in matrix microhardness and a decrease in eutectoid hardness values were observed, indicating an increase in the homogeneity of the heat-treated alloy. At the same time, an increase in scandium concentration in the alloy resulted in an increase in microhardness values of structural components both before and after heat treatment.

When increasing the holding time at temperatures of 150–250 °C, the microhardness of the alloys under study decreased due to a more complete decomposition of the eutectoid type δ + (MgZr)12Nd.

The addition of scandium to the alloy up to 0.07% contributed to an increase in both mechanical and heat resistance properties. Further, there was a tendency to decrease the physical and mechanical properties of the material.

Increasing the long-term strength test temperature to 270 °C reduced the time to failure by ~6 times. Samples with an additive of 1.0% Sc were destroyed when they were loaded already at 250 °C because of the formation of microloops and film impurities.

Thus, the scandium content in the metal in the range of 0.05–0.07% contributes to obtaining a finely dispersed, homogeneous structure and provides the best indices of mechanical properties and heat resistance of the alloy.

The developed composition of filler material was tested for the welding of magnesium castings. The structure and properties of the base metal and the weld were studied on the products welded with the developed scandium-containing filler material.

Some of the aircraft engine casings were welded with the use of the experimental filler material. After, they were cut for metallographic control and the production of samples for mechanical tests. Metallographic control of the welded hull elements showed that the weld metal and the base of the part had a dense homogeneous structure without defects. The microstructure of the base metal was characteristic of the heat-treated state; the weld consisted of a δ-solid solution and an intermetallic phase. At the same time, the size of the structural components in the weld was much smaller than in the base metal. To control the mechanical properties, samples were cut from the welded body parts. The samples were taken so that the boundary of the transition zone from the weld to the base metal was in the middle part of the samples. To determine this limit, samples were etched with a reagent consisting of 1% nitric acid, 20% acetic acid, 19% distilled water, and 60% ethylene glycol. X-ray control established good quality of welding. By the level of mechanical properties (σv = 230–235 MPa, δ = 3.5–4.0%), the examined metal exceeded the indices of the base metal, and in that specimen, destruction during tests was not along the weld but along the base metal.

Thus, the usage of scandium-containing filler material for welding complex hulls of aircraft engines made of a magnesium alloy of the Mg-Zr-Nd system allows for a high-quality weld and ensures the reliable operation of critical units. It is worth stressing that similar research is also being developed for others alloys, such as aluminum [45,46], and some obtained results could be an inspiration for others materials.

## 5. Conclusions

The application of scandium-containing filler material for welding products made of Mg-Zr-Nd alloy system helps improve significant mechanical properties, heat resistance, and the reliability of aircraft structures in general. Moreover, the developed technology for welding cast products made of magnesium alloy with scandium-containing filler enhances their operational characteristics and extends the service life of the aircraft mechanisms and units and results in a significant economic effect. The provided research allows us to formulate the following conclusions:

- The modification of the magnesium alloy with scandium in an amount of scandium between 0.05 and 0.06 of the mass allows us to obtain a fine-grained structure, an increase in the level of mechanical properties, and long-term strength at 250 °C due to the formation of complex intermetallic phases and a microalloying δ-solid solution.
- Welding of elements of cast parts from the alloy system, using Mg-Zr-Nd filler material containing scandium, enabled a dense homogeneous zone of alloying to be obtained with high mechanical properties.
- Industrial testing of the technological process of welding casting elements with scandium filler material allowed us to recommend it in the production process to obtain complex structures that meet operational regulatory requirements.

## Figures and Tables

**Table 1:** The elemental composition of used magnesium alloy.

Element	Mg	Zr	Nd	Zn
% by mass	96.0%	0.8%	2.6%	0.6%

**Table 2:** Microhardness of samples of magnesium alloy with different contents of Sc.

Amount of Sc (% by wt.)	Average Microhardness HV (MPa)			
	To Heat Treatment		After Heat Treatment	
	Matrix	Eutectic	Matrix	Eutectic
-	662.5	1916.6	1065.7	1320.4
0.02	770.0	2089.3	1078.8	1480.6
0.05	781.0	2094.9	1098.8	1504.7
0.07	827.1	2125.7	1114.4	1735.6
0.10	835.0	2158.7	1154.5	1891.6
0.30	846.5	2211.8	1187.4	1930.6
0.50	871.5	2285.9	1235.5	1985.7
0.70	904.3	2348.3	1288.4	2130.6
1.00	942.8	2450.7	1320.5	2211.6

**Table 3:** The obtained results of Micro-X-ray spectral analysis.

Element	Mg	Zr	Nd	Si	Sc
% by mass	93.52	1.83	4.0	0.08	0.57

**Table 4:** Average mechanical properties and long-term strength of magnesium alloy with different contents of Sc.

Amount of Sc (% by wt.)	Mechanical Properties		Long-Term Strength at σ = 80 MPa, Hours		
	σ_v_ (MPa)	δ (%)	T ^1^ = 150/250 °C	T = 270 °C	T = 300 °C
0	235.0	3.6	1252.0/2.,2	47.5	9.0
0.02	253.0	4.6	1252.0/56.0	53.1	11.1
0.05	245.0	6.3	1252.0/48.7	71.5	16.0
0.07	240.0	4.0	1252.0/64.0	61.6	12.4
0.10	232.0	3.5	1252.0/48.0	36.5	13.4
0.50	235.0	4.0	1251.0/34.1	24.0	6.7
1.00	169.0	3.3	1252.0/8.0	-	-

^1^ Tests of samples for long-term strength were performed in stages: at 150 °C (numerator), then at 250 °C (denominator).

**Table 5:** The average size of structural components, microhardness, and mechanical properties of welded samples of magnesium alloy.

Amount of Sc (% by wt.)	Average Size of Structural Components		Mechanical Properties	
	Matrix (μm)	Eutectic (μm)	σ_v_ (MPa)	δ (%)
0	70/34	40/30	235/239	3.6/3.2
0.05–0.06	60/25	45/35	245/253	5.6/6.0

Note: the numerator is the base metal, and the denominator is the weld.

## Data Availability

Not applicable.
